# Fatal hemorrhage from peripheral varicose vein rupture

**DOI:** 10.4322/acr.2021.330

**Published:** 2021-09-23

**Authors:** Guendalina Gentile, Stefano Tambuzzi, Michele Boracchi, Alessandro Del Gobbo, Paolo Bailo, Riccardo Zoia

**Affiliations:** 1 Università degli Studi di Milano, Dipartimento di Scienze Biomediche per la Salute, Sezione di Medicina Legale e delle Assicurazioni, Laboratorio di Istopatologia Forense e Microbiologia Medico Legale, Milano, Italy; 2 Fondazione IRCCS Ca' Granda - Ospedale Maggiore Policlinico Milano, Anatomia Patologica, Milano, Italy

**Keywords:** Autopsy, Hemorrhage, Varicose veins

## Abstract

Varix of the lower extremities is a common entity that eventually presents fatal outcome. Fatal massive bleeding due to rupture of a peripheral varicose vein is rare. The estimated incidence of these cases is 1/1000 autopsies. The case we present is unique among 26,054 autopsies performed in Milan from 1993 to 2020. It describes the investigations carried out in the suspicion of a non-natural event in an elderly woman. She was found dead at home with a large volume of blood near her feet that drained from the right leg. Pathological examination disclosed that the hemorrhage occurred by the rupture of a venous varix of the lower limb. Cases of fatal hemorrhage from peripheral variceal rupture are insidious and require proper characterization. The bloodstain pattern analysis, careful autopsy dissection by layers to demonstrate the rupture, and histologic examination of the lesion are the essential elements to find out the actual cause of death.

## INTRODUCTION

Chronic venous insufficiency (CVI) is present in 15-50% of the general population.^1^^,^ 2 It is rarely considered a life-threatening entity.^3^ The peripheral venous tortuosity is usually localized to the lower limbs.^4^ The risk factors associated with CVI are female gender, age, family history, obesity, and prolonged work activity in an orthostatic stance.^5^ Spontaneous or trauma-related^6^ rupture of the varix is an unusual complication.^3^^,^ 7 This eventual complication leads to an unexpected natural death due to severe bleeding and is documented in 0.15-2% of the population^,^ or in less than 1 in 1000 autopsy cases.^6^^,^ 8 ^-^ 12 The venous bleeding is distinguishable from the arterial bleeding^11^ by its proximity of the bleeding source due to the lower pressure of the venous system.^13^ However, in some cases, it may show a projected, scattered appearance when the pressure of blood in the distended veins is high.^13^ Thus, severe bleeding observed may raise misinterpretations and doubts about the injury mechanism.^1^^,^ 13 These cases require an often-challenging differential diagnosis between a non-natural cause of death and an accidental event.^6^^,^ 14 Our report discusses the challenges to understand the mechanism of the vessel injury and the cause of death on a single case of lethal rupture of the right lower limb varix in an older woman.

## CASE REPORT

A 91-year-old, self-sufficient, and lonely woman was found dead by the police; after two days, the neighbors did not see her. She was found sitting near the refrigerator with a pool of blood (measuring 87 cm in transverse diameter and 72 cm in longitudinal diameter) at her feet, despite other major blood traces scattered over the floor. Also, the victim's right hand showed traces of the same type. An already opened package of oral antiplatelet drugs (*Ticlid*, 250 mg) was found on the bedside table that the victim was eventually taking. The blood was localized and clustered in the hallway and near the chair where the deceased lady had been found. Also, a 2-euro metal coin smeared with blood on both sides was found. The pattern of blood flow lacked high-pressure projections; it was not scattered and did not show typical signs of high-pressure blood spillage (typically observed in arterial origin hemorrhage). This observation allowed us to consider it a venous type of bleeding. An autopsy 3 days after the discovery was undertaken by judicial request.

### Autopsy findings

**Figure 1:**
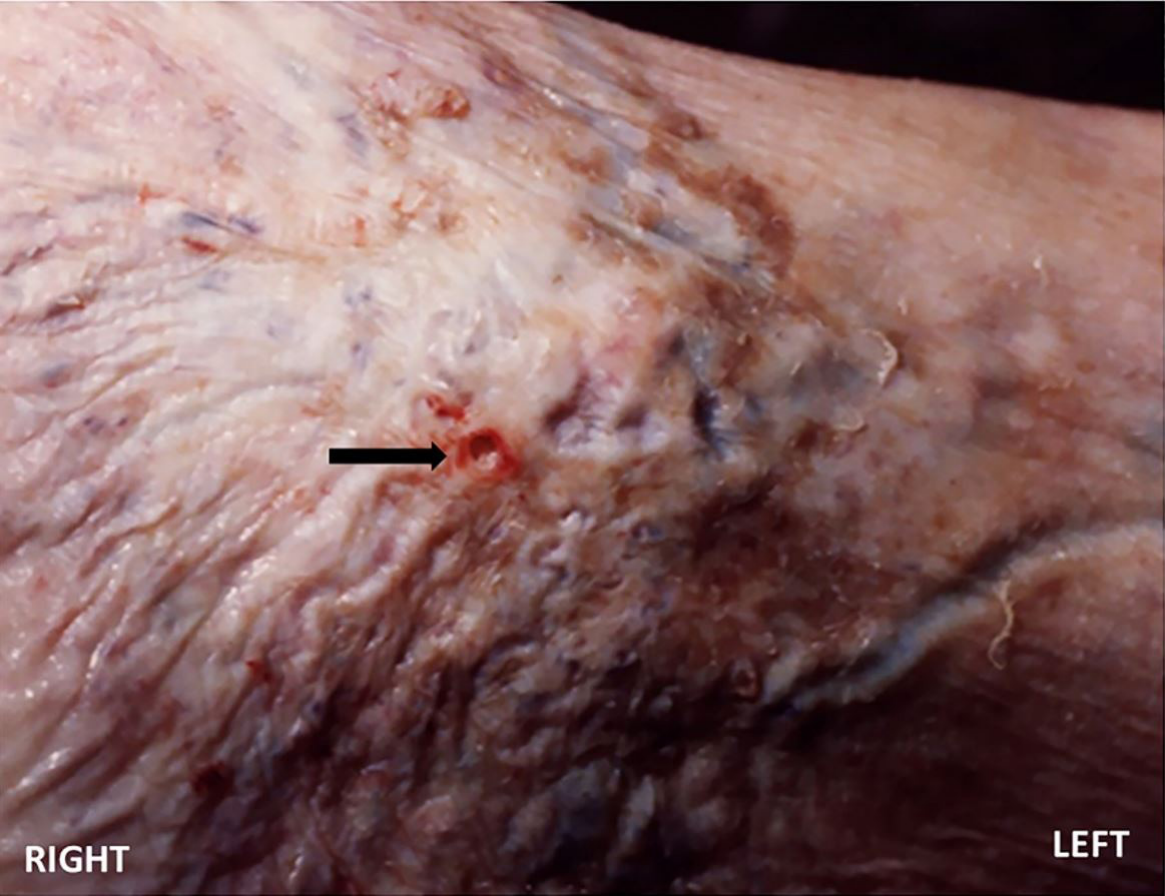
Right to left gross view of the venous tortuosity and break of the integument and venous wall (arrow).

On external examination, the corpse was in good conditions of nutrition and preservation. No signs of traumatic injuries or defense wounds were found. The hypostasis was reduced in intensity and extension. This finding was consistent with death due to acute hemorrhagic shock. The lower limbs showed clear signs of chronic venous disease and diffuse brownish dyschromic areas between 0.2 and 0.5 cm in diameter. A bluish serpiginous tegumentary swelling was observed at the distal third of the right leg on the anteromedial surface, and a minor full-thickness skin breach with the underlying venous wall was disclosed. The wound had irregular raised blood-stained margins, and it measured 0.5 x 0.3 cm (Figure 1).

The internal organs were diffusely pale. The brain parenchyma showed a yellowish and softened area in the left temporal area. Bilateral apical whitish pleural thickenings were documented, and reduced thickness of the renal cortex and atherosclerotic disease of the aorta and coronary arteries. There was no evidence of myocardial infarction. The only bleeding site was found in the right lower limb.

No other source of traumatic or natural bleeding was detected. After the autopsy, the cause of death was identified as acute hemorrhagic shock secondary to the rupture of a venous varix of the right lower limb. The histological analysis of the skin fragment taken from the suspected bleeding site was undertaken. The microscopic study of the remaining organs was not allowed.

### Microscopic features

**Figure 2:**
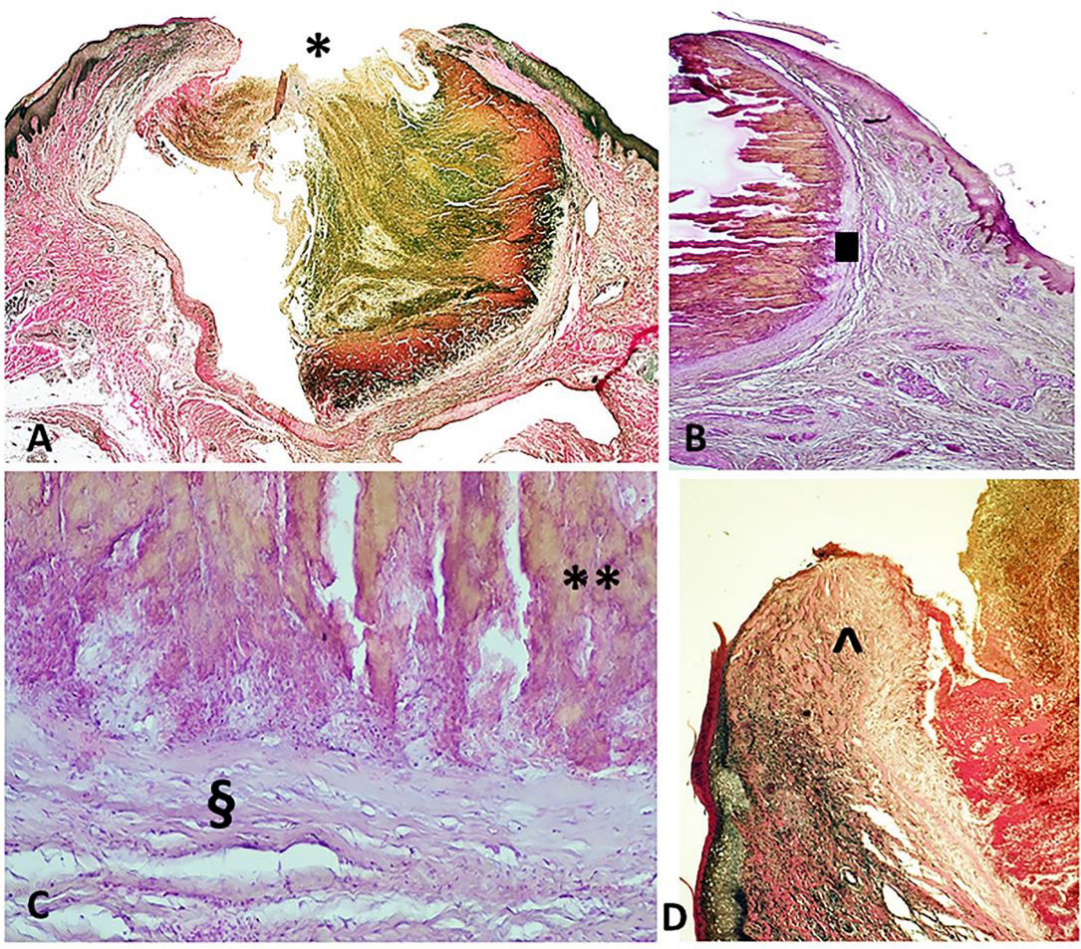
Photomicrographs of the vascular lesion. **A** – Panoramic view of the dermo-epidermal specimen including a cross-section of a superficial venous vessel at the point of rupture (*) with discontinuity of overlying tissues (van Gieson's trichrome, 2x); **B** – The subcutaneous soft tissue site of vascular ectasia of the venous origin. Note the lacking of muscular layer () and blood congestion (H&E, 20x); **C** – Phlebo-thrombotic material is characterized by numerous red blood cells alternating with layers of fibrin (Zahn's lines) (**) and initial adhesion/organization (§) (H&E, 40x); **D** – Granulation tissue (^) (van Gieson's trichrome, 10 x).

The skin fragment was processed with standard post-fixative histological examination. In the dermo-epidermal sample, the histological examination documented the cross-section of a markedly ectatic venous blood vessel, characterized by a thin wall and without smooth muscle fibers, filled with blood. The venous vessel was 80% occluded by a thrombotic material and was superficial to the skin layer at the point of rupture with discontinuity of the overlying tissues (Figure 2).

## DISCUSSION

The first reports of death from acute bleeding by peripheral varicose veins rupture date from the 1970s.^15^ Two major types of bleeding ulcers have been described. The first is acute perforated ulcers with small superficial lesions and adjacent pigmentation in surrounding healthy skin.^16^ The other type is chronic ulcerative ulcers (more extensive and more profound than the previous ones). This first description of acute varicose vein bleeding was followed by reports of spontaneous ruptures,^2^^,^ 3 ^,^ 17 or due to minor traumas.^18^ In the forensic field, the rare complication of fatal bleeding in subjects affected by limb venous varicosity^18^ is scarcely reported. The search performed on PubMed NCBI reports only 13 cases of pathological-forensic relevance.^1^^-^ 4 ^,^ 6 ^,^ 8 ^,^ 11 ^-^ 14 ^,^ 19 ^,^ 20 Many patients diagnosed with varicosities of non-severe degree underestimate the threaten of this entity. They consider it an aesthetic problem than a medical concern. However, this entity carries the potential threaten of massive and lethal bleeding. The time-lapse of death is between 5 and 20 minutes; despite the elevation of the affected limb and compression of the bleeding point seem easy maneuvers to save life.^21^ The risk of life-threatening rupture of peripheral varicosities is increased in the affected subjects that are socially vulnerable or in conditions of social isolation. This typically occurs in the elderly, who constitute the most exposed and vulnerable patients, because of their skin and soft tissue fragility.^11^ Therefore, more spontaneous and severe lesions are documented in these patients, even in apparently “intact” varices at the gross examination or after mild traumas than in younger people.^11^^,^ 12 The resulting profuse bleeding could be arrested with simple therapeutic hemostatic or adequate compression procedures.^10^ Yet the lack of awareness of the danger, the neglect, and/or the cognitive impairment may lead them to overlook a minor, continuous, and painless venous bleeding^11^ that, conversely, could prove to be unstoppable and, therefore, lethal.^19^

In the presented case, the 91-year-old lady lived alone. She had visible superficial peripheral varices and showed skin changes from chronic venous stasis. She was found in her home with a pool of blood near her feet which led the police to hypothesize a lethal injury despite the absence of signs of physical aggression such as fatal wounds. The only finding was the right lower limb was the single bleeding vascular lesion. Histologically, the latter was characterized as an ulcer of a very superficial dilated varicose vein, thin-walled and lacking smooth muscle and, as such, capable of predisposing to profuse bleeding.^12^ The bloodstain pattern analysis performed was consistent with venous bleeding, characterized by low pressure. The blood pool near the deceased was below the bleeding point from which it originated, which contributed to confirming the venous bleeding hypothesis.

We hypothesized two possible mechanisms as the cause of injury: a minor trauma, such as rubbing the right leg with an object, or a spontaneous rupture. We believe that the weakness of the ulcerated skin and vein wall contributed significantly to the rupture of the venous vessel. Regarding the presence of a 2-euro coin within the blood pool, we hypothesized that the patient tried to used it to stop the bleeding. Metal coins were considered an effective hemostatic means for small bleedings, according to a past popular tradition. Moreover, the chronic therapeutic use of oral platelet antiaggregant drug and the lack of assistance or request for help were further crucial factors for establishing a fatal bleeding condition.

This case of fatal hemorrhage from a peripheral varicose vein rupture as a cause of sudden death highlights the dangerous nature of chronic venous disease (which threat to life is neglected). It rapidly led to the end of life of a lonely and highly vulnerable older woman. The pathologist had to be careful before commenting on the cause and manner of death. The association of the blood pattern analysis and a thorough autopsy, the careful scene in which the deceased was found, together with the histological examination, represented the elements on which the final judgment was based. The actual incidence of such events is not yet fully known. It is, therefore, crucial to report these rare events because they undoubtedly can be a cause of sudden death.
