# ERRATUM: Fatal hemorrhage from peripheral varicose vein rupture

**DOI:** 10.4322/acr.2021.367

**Published:** 2022-03-28

Due to copyediting error the article “Fatal hemorrhage from peripheral varicose vein rupture” (DOI https://doi.org/10.4322/acr.2021.330), published in Autops. Case Rep, 11, 2021, was published with an error in the author's name in How to cite.

On How to cite, where the text reads:

How to cite: Gentile G, Tambuzzi S, Boracchi M, Gobbo A, Bailo P, Zoia R. Fatal hemorrhage from peripheral varicose vein rupture. Autops Case Rep [Internet]. 2021;11:e2021330. https://doi.org/10.4322/acr.2021.330

It should read:

How to cite: Gentile G, Tambuzzi S, Boracchi M, Del Gobbo A, Bailo P, Zoia R. Fatal hemorrhage from peripheral varicose vein rupture. Autops Case Rep [Internet]. 2021;11:e2021330. https://doi.org/10.4322/acr.2021.330

The publisher apologizes for the errors.

